# SUMOylation of FOXP1 regulates transcriptional repression via CtBP1 to drive dendritic morphogenesis

**DOI:** 10.1038/s41598-017-00707-6

**Published:** 2017-04-13

**Authors:** Daniel L. Rocca, Kevin A. Wilkinson, Jeremy M. Henley

**Affiliations:** School of Biochemistry, Centre for Synaptic Plasticity, Medical Sciences Building, University of Bristol, University Walk, Bristol, BS8 1TD UK

## Abstract

Forkhead Box P (FOXP) transcriptional repressors play a major role in brain development and their dysfunction leads to human cognitive disorders. However, little is known about how the activity of these proteins is regulated. Here, we show that FOXP1 SUMOylation at lysine 670 is required for recruiting the co-repressor CtBP1 and transcriptional repression. FOXP1 SUMOylation is tightly controlled by neuronal activity, in which synapse to nucleus signalling, mediated via NMDAR and L-type calcium channels, results in rapid FOXP1 deSUMOylation. Knockdown of FOXP1 in cultured cortical neurons stunts dendritic outgrowth and this phenotype cannot be rescued by replacement with a non-SUMOylatable FOXP1-K670R mutant, indicating that SUMOylation of FOXP1 is essential for regulation of proper neuronal morphogenesis. These results suggest that activity-dependent SUMOylation of FOXP1 may be an important mediator of early cortical development and neuronal network formation in the brain.

## Introduction

Forkhead Box P (FOXP) proteins are a subfamily of transcription factors that bind to promoter and enhancer sequences in many genes via a forkhead DNA binding motif to spatially and temporally control the expression of a wide range of genes^1^. FOXP family members are involved in embryonic morphogenesis^1^ and they play key roles in regulating the development and differentiation of cells in many tissue types. Furthermore, mutations in FOXP proteins have been implicated in many human developmental disorders^2^ and their malfunction can cause severe cognitive disorders^3, 4^. In humans, there are four members of the FOXP family, FOXP1-4 and, of these, FOXP1, FOXP2 and FOXP4 are highly expressed in the CNS, and have each been implicated in cortical development, and motor learning^3^. Mutations in FOXP2 cause verbal dyspraxia in humans^5^ and its deletion reduces vocalisaion in mice and impairs song learning in songbirds^6^.

Interestingly, FOXP1 and FOXP2 can form heterodimers to regulate transcription and they overlap in their expression pattern in songbird and foetal human brain, suggesting that FOXP1 may also have a role in speech and language disorders^7^. Moreover, mutations in FOXP1 are associated with intellectual disability and autism spectrum disorder (ASD)^8, 9^ and FOXP1 knockout mice display developmental deficits and reduced social interactions, similar to mouse models of autism^10^. Indeed, inhibition of *Foxp1* expression in the mouse cortex impairs neuronal migration, polarisation and the maturation of dendritic processes^10, 11^. However, how FOXP1 is regulated during early cortical development and the signals that initiate these processes remain unknown.

SUMOylation is a posttranslational modification that regulates the functions and fates of hundreds of proteins in nearly all cell pathways^12^ and in the brain it directly impacts on diverse aspects of neuronal morphology and function^13^. The consequences of SUMOylation are varied but the underlying principle is that it alters inter- and/or intramolecular interactions to change substrate protein localisation, stability, and/or activity. Furthermore, inappropriate regulation of the SUMO pathway is a common factor in many neurological and neurodegenerative diseases^13^. Both FOXP1 and FOXP2 bind SUMO E3 ligases, and have been demonstrated to be SUMO substrates^14^. SUMOylation of cerebellar FOXP2 in mouse neonates modifies its transcriptional activity to regulate Purkinje cell development, dendritic outgrowth and arborization, and is required for correct cerebellar motor function and vocal communication^15, 16^.

Here we demonstrate that endogenous FOXP1 is SUMOylated at K670 in rat neurons and this is required for efficient transcriptional repression, likely by promoting binding to the transcriptional co-repressor CtBP1. FOXP1 SUMOylation is activity-dependently regulated via calcium entry through both NMDA receptors (NMDARs) and L-type calcium channels. Ablation of FOXP1 in cultured cortical neurons dramatically stunts dendritic outgrowth and this phenotype can be rescued by expression of wild-type but not a non-SUMOylatable FOXP1-K670R mutant. Moreover, we show that FOXP1 SUMOylation is required for regulation of *CNTNAP2* expression, a candidate autism gene involved in dendritic maturation. Thus, FOXP1 SUMOylation is essential for the proper neuronal morphogenesis that ultimately is required for cortical development and neuronal network formation.

## Results

### FOXP1 is SUMOylated at K670

**Figure 1:**
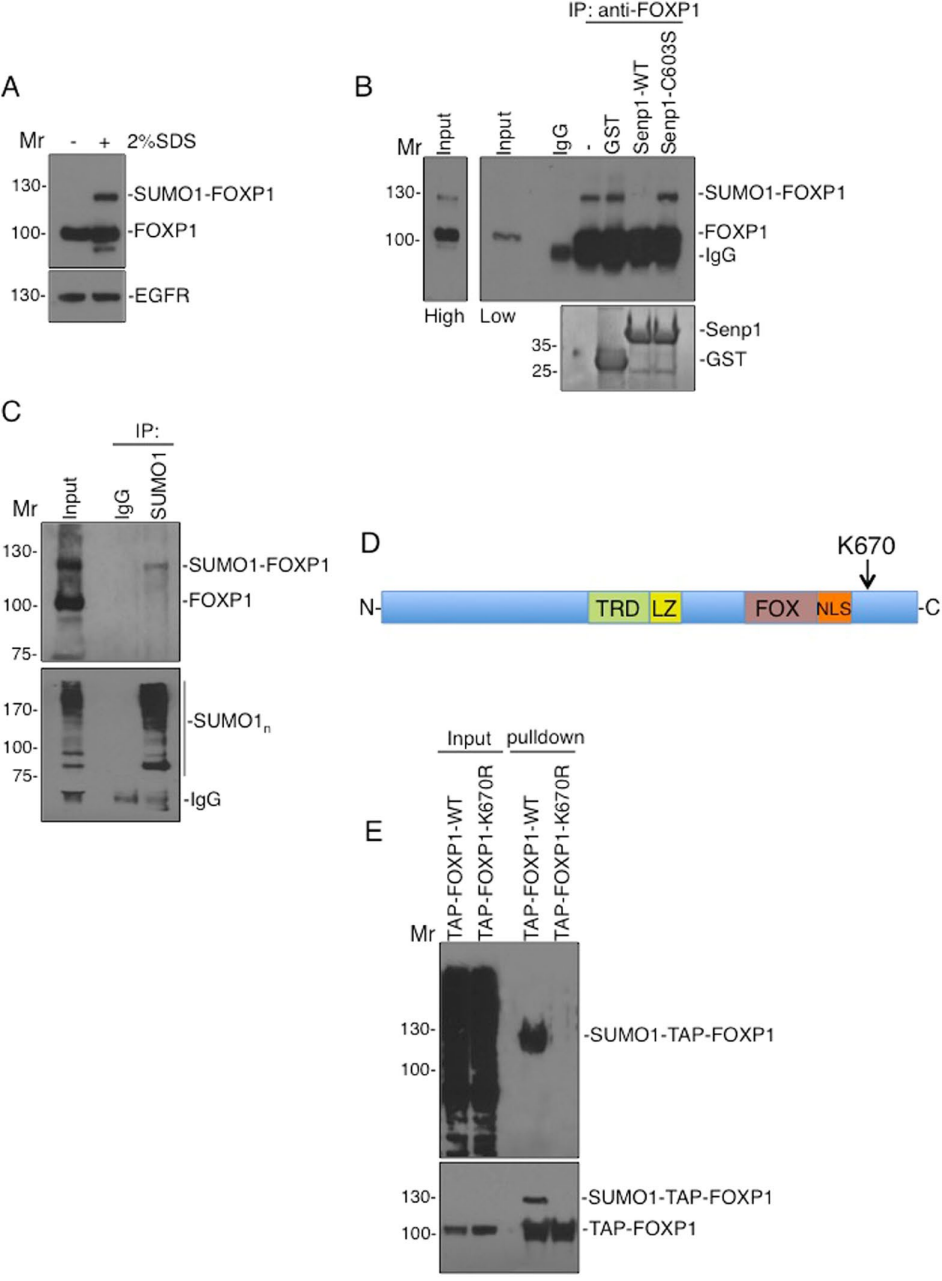
FOXP1 is SUMOylated at K670. (**A**) DIV7 cortical neuronal cultures were lysed in buffer with or without 2% SDS and immunoblotted with FOXP1 antibody. Under denaturing conditions a prominent additional higher molecular weight FOXP1 band was observed. (**B**) Following lysis in 2% SDS containing buffer, the SDS was diluted to 0.1% and FOXP1 immunoprecipitated and incubated with GST, GST-WT-Senp1 catalytic domain or GST-Senp1-C603S catalytic domain. Immunoblots with FOXP1 antibody (top panel) shows the higher Mr band is removed by Senp1 treatment consistent with it being SUMOylated FOXP1. A high exposure (top left panel) of the input shows the presence of the modified-FOXP1. Lower panel shows equal levels of purified GST-SENP proteins by coomassie staining. (**C**) Immunoprecipitation was performed as in B, using a SUMO-1 antibody, followed by immunoblotting with either FOXP1 (upper panel) or SUMO1 (lower panel) antibodies. SUMO1-modified FOXP1 corresponds to the high Mr band in the input. Note the enrichment of SUMO1-ylated proteins (seen as a smear) in the IP lane but not the control IgG. (**D**) Schematic of FOXP1 showing the putative SUMOylated lysine. TRD, transcriptional repression domain; LZ, leucine zipper; FOX, FOX homolgy domain; NLS, nuclear localisation signal. (**E**) FOXP1 is SUMOylated at lysine 670. HEK293T cells were transfected with either TAP-FOXP1-WT or the mutant TAP-FOXP1-K670R, lysed in buffer containing NEM, to inhibit SUMO proteases, and precipitated using strepavidin beads before subsequent immunoblotting with anti-SUMO1 (top panel) or anti-SBP (lower panel) antibodies.

We initially determined if FOXP1 is a target for SUMOylation in neurons by lysing primary cultured cortical neurons under either native or strong denaturing conditions (2% SDS) to prevent SUMO deconjugation by SUMO proteases. Western blots for FOXP1 displayed a very robust higher molecular weight species under denaturing conditions, with a band shift consistent with a SUMOylated form of the protein (Fig. 1A). To confirm that the higher molecular weight band constituted SUMOylated FOXP1 we immunoprecipitated FOXP1 and incubated the immunoprecipitate with active or inactive catalytic domains of the SUMO-specific protease Senp1. The higher molecular weight band was absent in the active Senp1 samples but present in the samples treated with the inactive point mutant (Senp1-C603S; Fig. 1B). Additionally, immunoprecipitation of SUMOylated proteins with SUMO1 antibody and immunoblotting for FOXP1 also detected a band corresponding to SUMOylated FOXP1 (Fig. 1C). These data confirm that FOXP1 is strongly SUMO1-ylated under basal conditions in cultured rat cortical neurons.

SUMOylation often occurs at lysines in the consensus sequence ΨKX(D/E)^17, 18^. Using a prediction algorithm (SUMOplot)^19^ we identified a single lysine (K670) as a high-confidence SUMOylation site (Fig. 1D). To determine if this is the SUMOylation site we cloned rat FOXP1-WT and a non-SUMOylatable mutant (FOXP1-K670R) into a tandem affinity purification (TAP) tagging vector. When expressed in HEK293T cells TAP-FOXP1-WT was robustly SUMOylated whereas there was no SUMOylation of TAP-FOXP1-K670R (Fig. 1E) indicating that lysine 670 is the sole SUMOylation site in FOXP1.

### SUMOylation of FOXP1 promotes transcriptional repression

**Figure 2:**
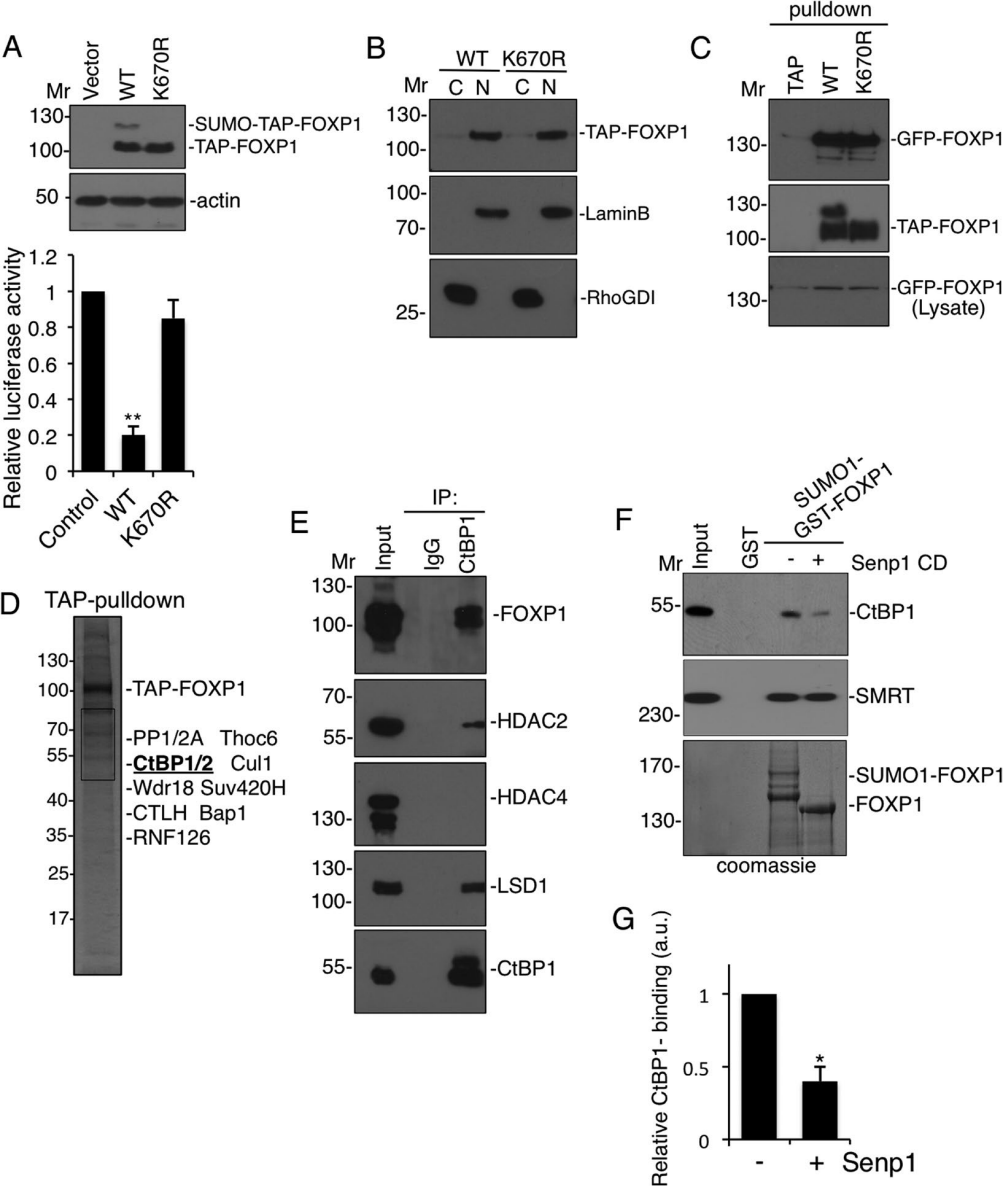
SUMOylation of FOXP1 regulates transcriptional repression and co-repressor binding. (**A**) SV40-driven luciferase was transfected into HEK293T cells along with either empty TAP vector, TAP-FOXP1-WT or TAP-FOXP1-K670R. Firefly luciferase activities were measured and normalised to Renilla lucifierease 48 hrs after transfection. Student’s t-test, n = 4, **p < 0.01 Note equal expression of TAP-tagged WT or K670R mutant in HEK293T cells (upper panels). (**B**) HEK293T cells were transfected with either TAP-FOXP1-WT or TAP-FOXP1-K670R, fractionated into cytosolic (**C**) or nuclear fractions (N) and immunoblotted. No differences were observed indicating that SUMOylation does not regulate nuclear localisation of FOXP1. (**C**) Empty TAP vector, TAP-FOXP1-WT or TAP-FOXP1-K670R were cotransfected into HEK293T cells with GFP-FOXP1. The cells were lysed, TAP-FOXP1 variants precipitated with strepavidin beads, and immunoblotted revealing that SUMOylation does not affect FOXP1-dimerization. Lower panel shows equal expression levels of GFP-WT-FOXP1 across conditions. (**D**) TAP-FOXP1 was expressed in HEK293T cells, precipitated using strepavidin beads and resolved by SDS-PAGE followed by silver staining. Gel slices containing novel bands compared to control empty vector pulldowns (not shown), were trypsinised and proteins identified using Orbitrap mass spectrometery. (**E**) Cortical neuronal cultures (DIV 7) were lysed and immunoprecipitated with anti-Ctbp1 antibodies before being resolved using SDS-PAGE and immunoblotted as indicated. (**F**) SUMOylation of FOXP1 modulates Ctbp1 corepressor binding. Bacterially-purified SUMOylated GST-FOXP1 was bound to glutathione sepharose beads in the presence or absence of the Senp1-catalytic domain to mediate deSUMOylation. DeSUMOylated GST-FOXP1 is observed as a single band in the lower panel at around 130 KDa compared to the larger SUMOylated GST-FOXP1 bands (Lower panel). The beads were then incubated with lysates from cultured cortical neurons. Samples were resolved by SDS-PAGE and blotted for Ctbp1 or SMRT. (**G**) Quantification of (F) is shown as relative Ctbp1 binding to FOXP1. Student’s t-test, n = 3, *p < 0.05.

We next tested how SUMOylation regulates FOXP1 function. The SV40 promoter is a well-characterised target for FOXP1 repression^20^ so we co-transfected HEK293T cells with a reporter plasmid containing an SV40-driven firefly luciferase together with either empty TAP vector, TAP-FOXP1-WT or TAP-FOXP1-K670R. TAP-FOXP1-WT reduced luciferase activity by ~80% whereas TAP-FOXP1-K670R did not significantly repress SV40-driven luciferase expression (Fig. 2A) demonstrating that SUMOylation is required for effective FOXP1 transcriptional repression.

### SUMOylation does not regulate the localisation, stability or dimerization of FOXP1

SUMOylation often alters the localisation and/or stability of target proteins so we compared the nuclear localisation and total levels of TAP-FOXP1-WT or TAP-FOXP1-K670R. There was no difference between the nuclear and cytosolic localisation or total protein levels of WT and non-SUMOylatable FOXP1 in transfected HEK293T cells (Fig. 2B). Because FOXP proteins need to dimerise to be transcriptionally active^21^ we tested if SUMOylation is required for FOXP1 dimerisation. Both TAP-FOXP1-WT and TAP-FOXP1-K670R efficiently homodimersised with GFP-tagged FOXP1 (Fig. 2C) indicating that SUMOylation does not control nuclear partitioning, stability or dimerization of FOXP1.

### SUMOylation of FOXP1 enhances binding to the co-repressor Ctbp1

A prime role of SUMOylation is to alter protein-protein interactions so we reasoned that SUMOylation could regulate protein complex assembly required for FOXP1 activity. To find novel FOXP1-interacting proteins we expressed either empty TAP vector or TAP-FOXP1-WT in HEK293T cells followed by pulldown on streptavidin beads and analysed proteins specifically retained by the TAP-FOXP1 matrix via mass spectrometry. We identified multiple binding proteins (Fig. 2D) and, of these, we focused on C-terminal-binding protein 1 (CtBP1)^22^, which is a transcriptional co-repressor for FOXP proteins^20^.

We validated the proteomics data by confirming that FOXP1 and the known Ctbp1 co-repressor complex components histone lysine demethylase LSD1^23^ and HDAC2^24^ co-immunoprecipitated with Ctbp1, whereas the class IIa histone de-acetylase HDAC4, which is not normally associated with this complex, did not (Fig. 2E).

We then tested the effect of FOXP1 SUMOylation on recruitment of the Ctbp1 co-repressor complex using glutathione sepharose beads coupled to recombinant, bacterially-produced SUMOylated or non-SUMOylated FOXP1^25^. Beads were incubated with neuronal lysates and blotted for Ctbp1 and the co-regulatory protein SMRT, a known FOXP1 interaction partner^26^. Intriguingly SMRT bound equally to both SUMOylated and non-SUMOylated FOXP1 whereas Ctbp1 binding to non-SUMOylated FOXP1 was dramatically diminished (Fig. 2F,G). Thus, FOXP1 SUMOylation specifically recruits Ctbp1 and reduced Ctbp1 recruitment presumably accounts for the lack of transcriptional repression by FOXP1- K670R.

### Depolarisation decreases FOXP1 SUMOylation

**Figure 3:**
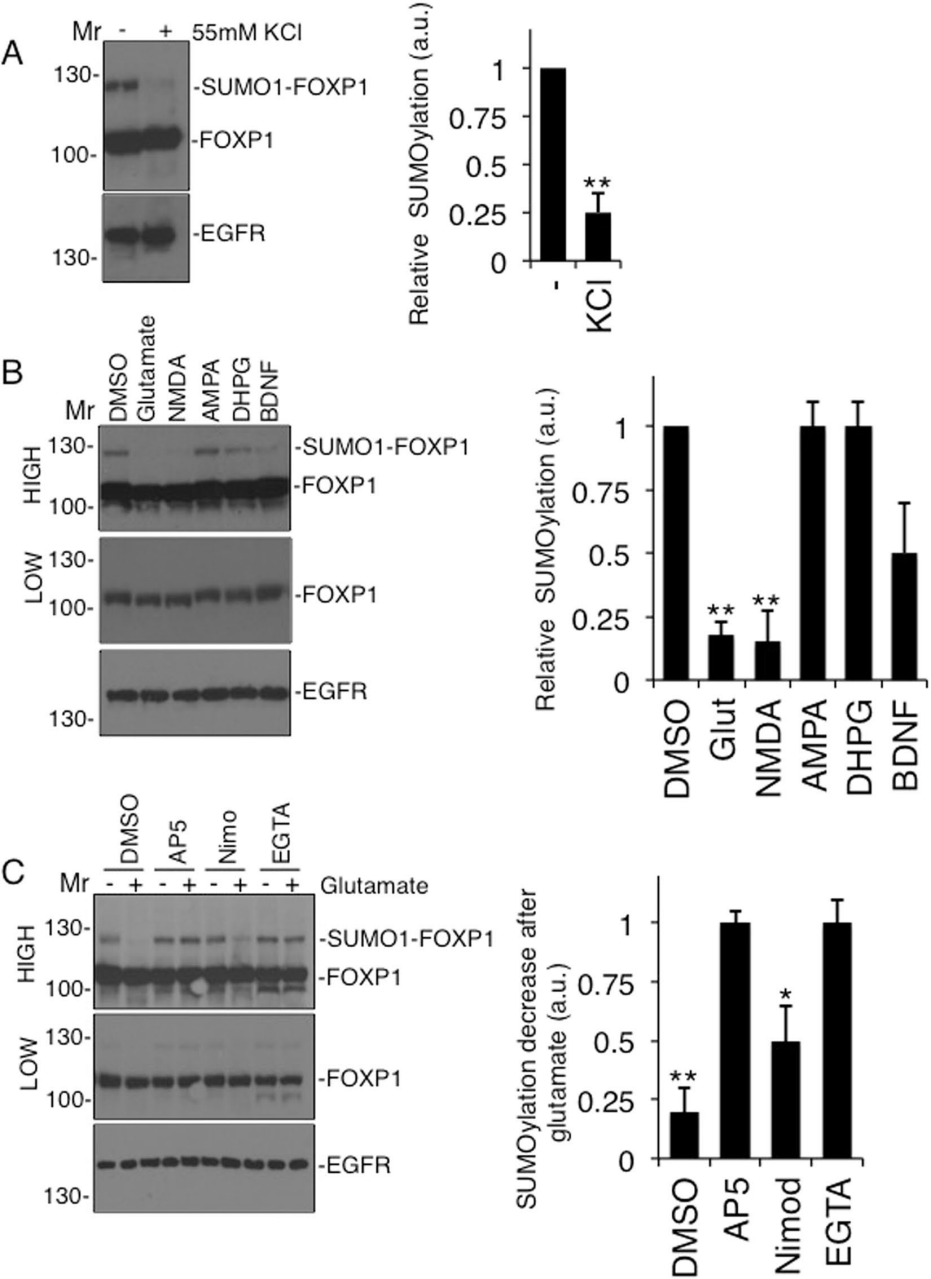
Activity-dependent regulation of FOXP1 SUMOylation. (**A**) Cultured cortical neurons (DIV7) were depolarised for 30 min with KCl in ACSF, lysed in sample buffer and resolved by SDS-PAGE followed by immunoblotting with the indicated antibodies. Student’s t-test, n = 4, **p < 0.01. (**B**) Cortical neurons were treated with the indicated drugs, lysed in sample buffer and samples resolved by SDS-PAGE and immunoblotting. NMDA receptor activation decreases FOXP1 SUMOylation. Note the lower exposure in the middle panel indicates unchanged expression levels of unmodified FOXP1. Student’s t-test and Welch correction, n = 5 **p < 0.001. (**C**) Calcium entry through NMDARs and L-type calcium channels reduces FOXP1 SUMOylation. Cultures were pre-treated with the NMDAR antagonist AP5, the L-type calcium channel blocker nimodipine or EGTA to chelate extracellular Ca^2+^ prior to glutamate stimulation. Data normalised to no glutamate, DMSO control. ANOVA, n = 5, **p < 0.01, *p < 0.05.

Because neuronal maturation, cortical development and network formation are largely driven by neuronal activity^27, 28^ we next examined whether FOXP1 SUMOylation is regulated by neuronal activity. Depolarising neurons with 55 mM KCl dramatically reduced FOXP1 SUMOylation (Fig. 3A). To investigate possible pathways leading to this depolarisation-evoked reduction we tested a range of excitatory receptor agonists (Fig. 3B). Only treatment with glutamate and NMDA elicited a significant decrease in FOXP1 SUMOylation (Fig. 3B). Indeed, the NMDA receptor (NMDAR) antagonist AP-5 prevented the glutamate-evoked decrease in FOXP1 SUMOylation (Fig. 3C) whereas AMPA, the metabotropic glutamate receptor agonist DHPG, and the neurotrophin BNDF had no significant effect (Fig. 3B), strongly suggesting neuronal activity regulates FOXP1 SUMOylation through NMDARs.

**Figure 4:**
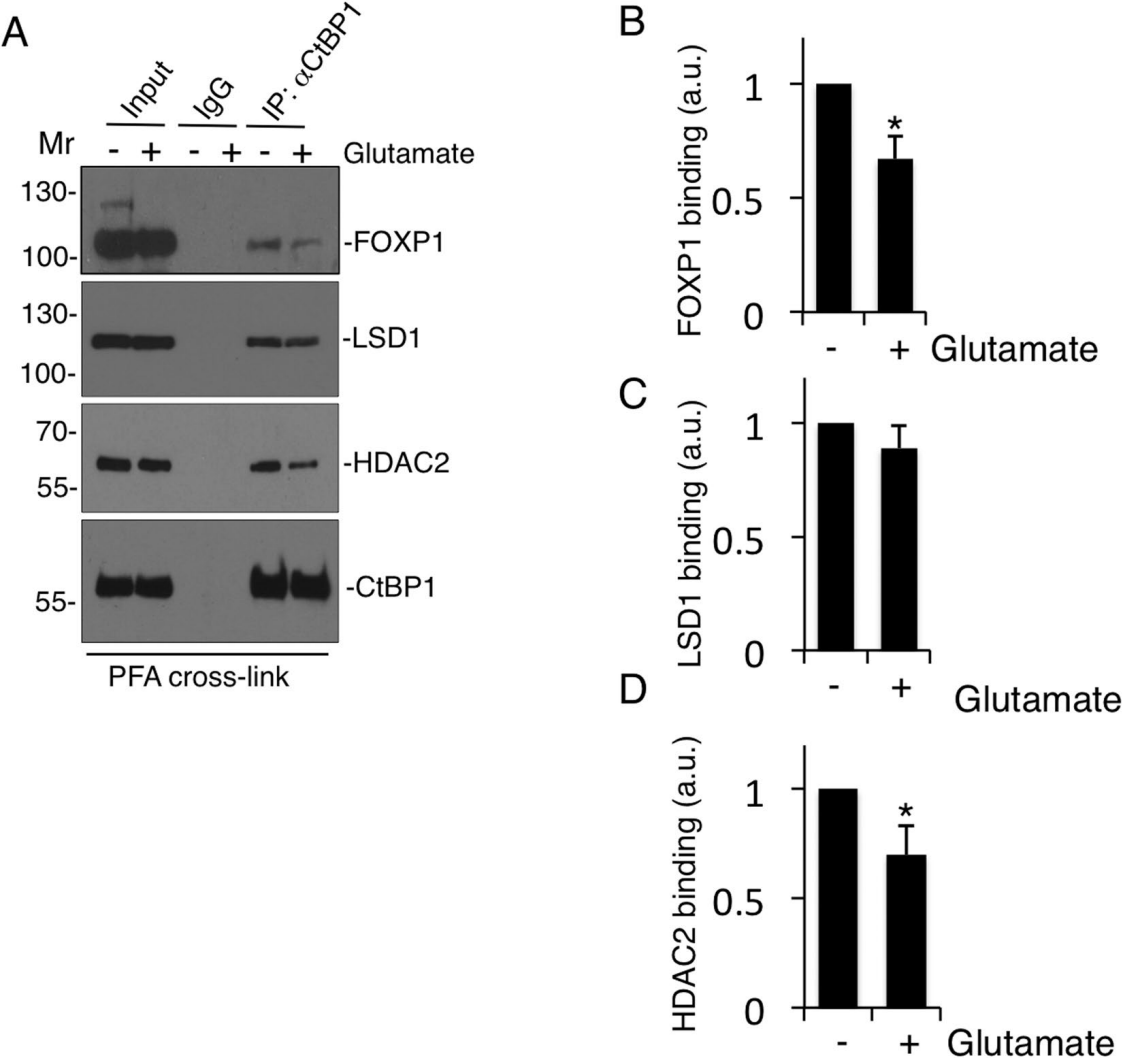
Activity-dependent modulation of FOXP1 recruitment to CtBP1 co-repressor complexes. (**A**) Glutamate receptor activation inhibits FOXP1 binding to Ctbp1. Co-immunoprecipitation of Ctbp1 complex and FOXP1 following glutamate stimulation of cultured cortical neurons was preceded by a fixation step using PFA to prevent deSUMOylation followed by immunoblotting with FOXP1, Ctbp1, LSD1 and HDAC2 antibodies. (**B**) Quantification of FOXP1 binding as shown in (**A**) shows marked reduction in the Ctbp1-FOXP1 interaction. Student’s t-test, n = 3, *p < 0.05. (**C**) Histogram of LSD1 binding as shown in (**A**) shows no-change in the Ctbp1-LSD1 interaction. (**D**) Quantification of HDAC2 binding as shown in (**A**) shows a decrease in the Ctbp1-HDAC2 interaction. Student’s t-test, n = 3, *p < 0.05.

A major feature of NMDARs is that they gate Ca^2+^ so we tested if extracellular Ca^2+^ is required for the decrease in FOXP1 SUMOylation using the chelator EGTA. Consistent with a signalling pathway involving the influx of extracellular Ca^2+^ through NMDARs leading to nuclear changes^29^, EGTA prevented the glutamate-mediated decrease in FOXP1 SUMOylation (Fig. 3C). The L-type calcium channel inhibitor nimodipine also reduced the glutamate-mediated reduction in FOXP1 SUMOylation, albeit to a lesser extent than AP-5 (Fig. 3C). Furthermore, in line with minimal Ctbp1 binding to non-SUMOylated FOXP1 (Fig. 2F), glutamate stimulation also reduced the Ctbp1-FOXP1 interaction (Fig. 4A,B) providing a mechanistic link between neuronal activity and FOXP1-mediated transcriptional repression. Interestingly, glutamate stimulation also caused a decrease in CtBP1 binding to HDAC2 but not to LSD1 (Fig. 4C,D), suggesting neuronal activity can result in a partial disassembly of co-repressor complexes in addition to regulating FOXP1 recruitment. Taken together, these results suggest that FOXP1-SUMOylation is highly regulated by activity-dependent signalling pathways that impact on gene-regulatory pathways involved in neuronal morphogenesis^29^.

### FOXP1 expression is developmentally regulated

**Figure 5:**
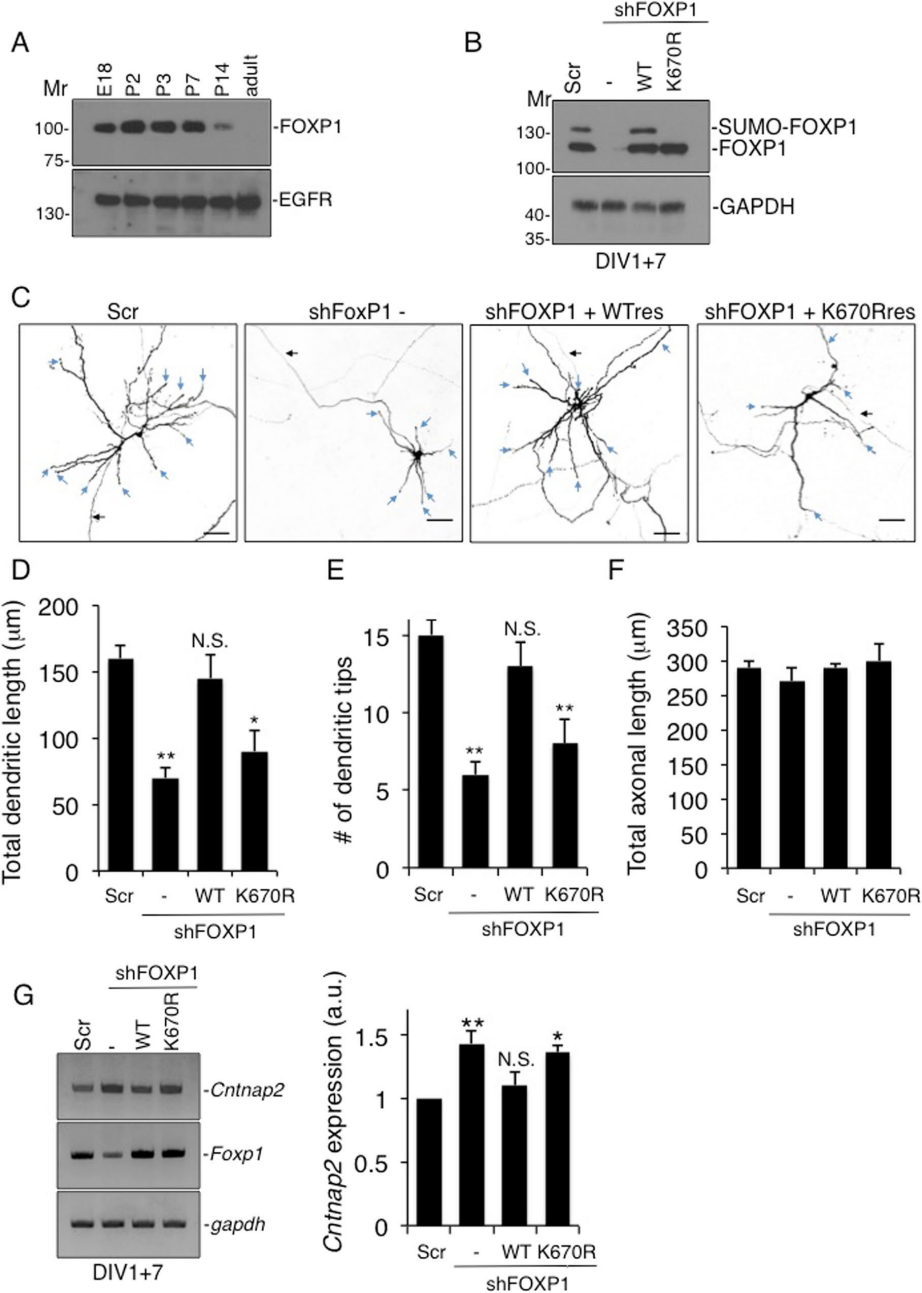
SUMOylation of FOXP1 regulates dendritic outgrowth and length. (**A**) Expression of FOXP1 peaks during early development. Non-denaturing lysates of either embryonic or postnatal brain cortices were analysed by SDS-PAGE and immunoblotting. (**B**) Cortical neurons lentivirally-infected with the indicated knockdown or knockdown plus FOXP1-WT or FOXP1-K670R rescue constructs at DIV1 were cultured for a further 7 days and then lysed in sample buffer and resolved by immunoblotting. (**C**) SUMOylation of FOXP1 is required for dendritc outgrowth. DIV1 cultured cortical neurons were transfected with constructs expressing either scrambled shRNA, FOXP1 shRNA or FOXP1 shRNA plus FOXP1-WT or FOXP1-K670R rescue and prepared for imaging at DIV8. In all images of neuronal morphology, blue arrows and black arrowheads indicate dendrites and axons, respectively. Scale bar, 10 μm. (**D**) Knockdown of FOXP1 significantly shortens dendritic length. FOXP1-WT but not the SUMOylation-deficient FOXP1-K670R rescued the dendritic phenotype. 312 neurons were analysed using the neuron J plugin for Image J. ANOVA, **p < 0.01, *p < 0.05. (**E**) Knockdown of FOXP1 significantly reduces the total number of dendritic tips. FOXP1-WT but not the SUMOylation-deficient FOXP1-K670R rescued the deficit in dendritic tip number. 302 neurons were analysed using the neuron J plugin for image J. ANOVA, **p < 0.01. (**F**) Axonal length was not significantly different between any FOXP1 knockdown or rescue with either WT-FOXP1 or the SUMOylation-deficient K670R FOXP1 mutant. (**G**) Knockdown of FOXP1 significantly increases *Cntnap2* expression. DIV1 cultures were infected as in C, followed by RNA extraction and RT-PCR analysis. FOXP1-WT but not the SUMOylation-deficient FOXP1-K670R rescued *Cntnap2* transcript levels back to control levels. ANOVA, n = 4, **p < 0.01, *p < 0.05.

Since the reported phenotypes of FOXP1 mutations are largely associated with neurodevelopmental defects^10^ we investigated the profile of FOXP1 expression in rat cortex (Fig. 5A). FOXP1 is highly expressed at E18 and up to postnatal day 7 but declines rapidly thereafter, is only weakly expressed at P14 and is absent in adult brain. This developmental profile corresponds to the period of intense neuronal maturation that drives neuronal network formation^30^.

### FOXP1 SUMOylation is essential for normal dendrite development

To determine how FOXP1 SUMOylation in cortical neurons affects differentiation, we used virally-expressed shRNAs to ablate endogenous FOXP1 and replace it with shRNA-insensitive FOXP1-WT or FOXP1-K670R. As proof of concept, we infected cortical neurons at DIV1 and analysed them at DIV7. As anticipated, FOXP1 knockdown at DIV1 markedly reduced levels of endogenous FOXP1 (Fig. 5B). Furthermore, at DIV7 endogenous and recombinantly expressed FOXP1-WT were robustly SUMOylated but there was no detectable SUMOylation of FOXP1-K670R (Fig. 5B).

To examine the role of FOXP1 SUMOylation on neuronal morphology, we next transfected DIV1 cortical neurons with constructs expressing either scrambled shRNA, FOXP1 knockdown, or FOXP1 knockdown with WT or K670R ‘rescue’ and assessed neurons at DIV8. FOXP1 ablation caused severely stunted dendritic development and reduced dendritic length by ~50% (Fig. 5C). Replacement with FOXP1-WT rescued this phenotype but non-SUMOylatable FOXP1-K670R did not (Fig. 5C,D). FOXP1 knockdown also reduced the number of dendritic tips by ~50% (Fig. 5E), suggesting a role for FOXP1 in branching of the dendritic tree. Again, this phenotype was rescued by WT-FOXP1 but by the K670R mutant. Total axonal length was unaffected in any of the conditions (Fig. 5F). These data demonstrate a specific role for tonic SUMOylation of FOXP1 in normal cortical neuron development and morphogenesis.

Although there are likely to be many neuronal-specific transcriptional targets of FOXP1 repression responsible for regulating dendrite morphogenesis, one validated target is the candidate autism gene *CNTNAP2* ^31^. Since *Cntnap2* plays a key role in dendrite arborization^32^, we tested whether FOXP1-SUMOylation is required for efficient transcriptional repression of the *rat Cntnap2* gene. FOXP1 knockdown caused a marked increase in *CNTNAP2* expression, presumably by relieving repression of its transcription (Fig. 5G). Expression of shRNA-resistant WT FOXP1 in the knockdown cells returned *Cntnap2* to near baseline levels. However, consistent with SUMOylation being required for efficient transcriptional repression, replacement with non-SUMOylatable FOXP1-K670R did not reduce the increased levels of expression observed in the FOXP1 knockdown cells.

## Discussion

Here we show that SUMOylation of the transcriptional repressor FOXP1 is a key regulator of normal dendritic outgrowth and complexity in the brain. In agreement with a recent study^11^ we find that FOXP1 expression in rat cortex is developmentally regulated and FOXP1 is maximally expressed when neurons are maturing and forming neuronal networks. FOXP1 SUMOylation acts as an interaction platform for recruitment of the CtBP1 co-repressor complex and is regulated by activity-dependent Ca^2+^ signalling. This fits well with the established role of SUMO modification in regulating the protein-protein interactions of transcription factors^33^ and uncovers a novel mechanism of neuronal activity ‘fine-tuning’ transcriptional co-repressor recruitment through SUMOylation. Moreover, it has been proposed that spatially confined proteins can be simultaneously and synergistically SUMOylated to facilitate protein complex formation^34^. Consistent with this concept the FOXP1 ‘co-repressor’ components CtBP1^35^ and HDAC1/2^36^ are targets for SUMOylation. Furthermore, neuronal activity results in the loss of HDAC2 from the CtBP1 corepressor complex, suggesting that SUMOylation may also participate in the regulation of core corepressor assembly.

Our finding that tonic FOXP1 SUMOylation selectively regulates dendrites but not axons suggests that active FOXP1 is part of the cell-intrinsic transcriptional program that controls dendritic specification in the cortex^37^. This is also consistent with SUMOylation playing a general role in dendritic morphogensis^38, 39^. Activity regulates both positive and negative mediators of dendritic complexity in neurons^40^ and the activity-dependent signalling that modulates FOXP1 SUMOylation provides further evidence that FOXP1 is both integral to, and tightly regulated during, neuronal development. In apparent contrast, however, a recent study using FOXP1 knockout mice reported that FOXP1 regulates axonal length and neuronal migration in the cortex^11^. While still unclear, reasons for this discrepancy may involve differences between the effects of chronic knockout in mice and acute shRNA-mediated knockdown of FOXP1 in rat neurons.

**Figure 6:**
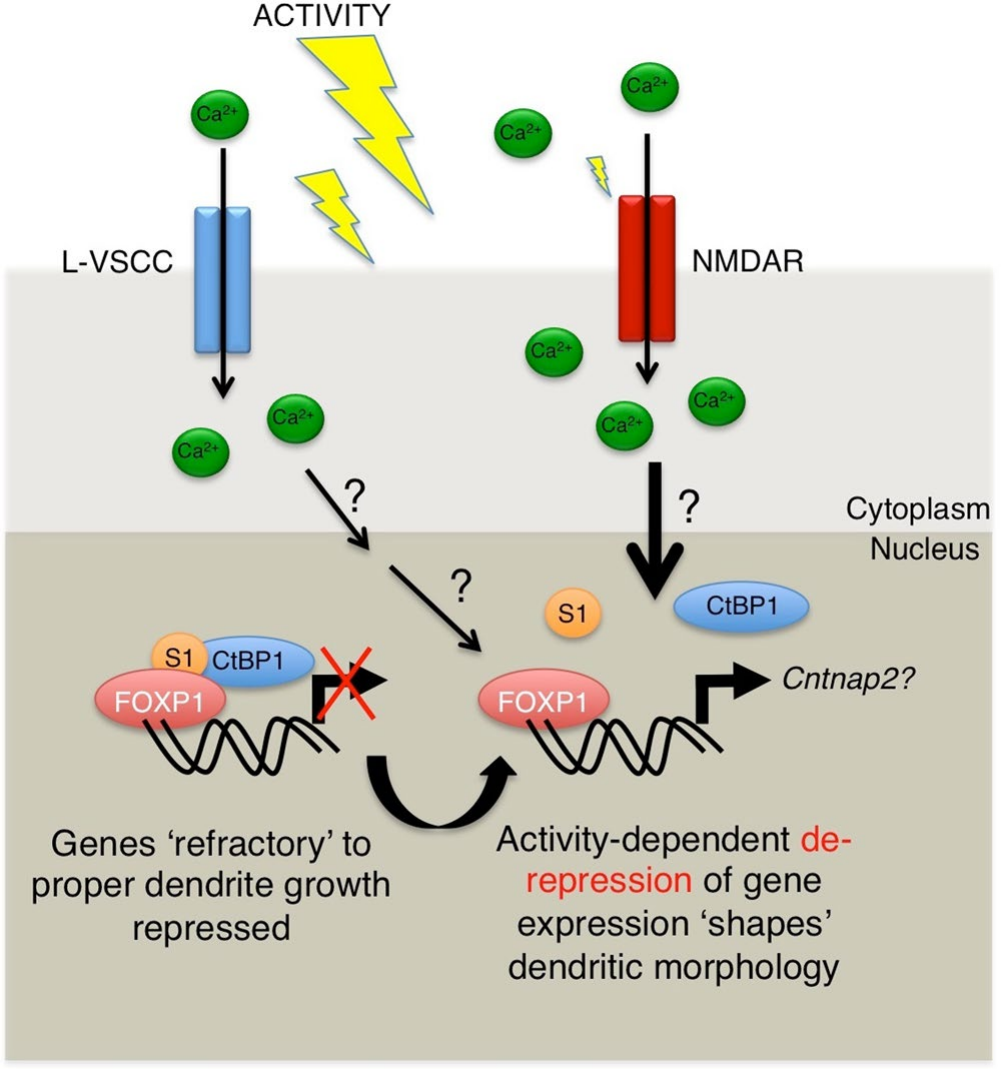
SUMOylation of FOXP1 regulates dendritic outgrowth and length. Taken together, our data suggests a model in which activity-dependent signals trigger the dynamic reduction of FOXP1 SUMOylation, as well as co-repressor recruitment, thereby modulating its function as a crucial transcriptional regulator that tonically controls the normal developmental maturation of neurons. We believe timely deSUMOylation of FOXP1 allows neurons to appropriately convert such activity-dependent cues into programs of gene-expression that finely ‘shape’ and ‘mature’ the dendritic tree (as opposed to continual growth) to aid and guide the structural and functional maturation of neuronal circuits.

We propose a model of the activity-dependent regulation of FOXP1 function during dendrite development (Fig. 6). SUMOylated FOXP1 acts as a crucial transcriptional regulator that tonically represses key target genes to control the normal developmental maturation of neurons. How then can activity-dependent signals also lead to dendritic growth? We suggest that temporally controlled deSUMOylation of FOXP1 can direct dendritic outgrowth to appropriately integrate activity-dependent cues into programs of perinatal gene expression that shape and mature dendritic arborisation.

Although FOXP1 is only highly expressed around birth, the constant modulation of FOXP1 SUMOylation by activity-dependent signals will facilitate the conditions for shaping the dendritic tree and establishing the genetic landscape required for neuronal maturation, prior to loss of FOXP1 expression and set the scene for subsequent refinement of arborisation by other mechanisms later in development.

Given that SUMOylated FOXP1 must, in part, control the expression of genes that are required for normal dendritic morphogenesis a key, as yet unanswered, question is what the identity of these genes might be? The autism-linked, neurexin-like adhesion molecule CNTNAP2 (also known as CASPR2)^41^ is a strong candidate. CNTNAP2 is required for dendritic arborisation^32^ and spine development^42, 43^. Both FOXP1 and FOXP2 repress CNTNAP2 expression^31, 44^ and in knockdown rescue experiments we show that non-SUMOylatable FOXP1 fails to repress *Cntnap2* expression (Fig. 5G). Together, these data suggest that SUMOylated FOXP1 normally represses expression of *Cntnap2* but that temporally enhanced expression mediated by FOXP1 deSUMOylation may be required to facilitate appropriate dendritic growth and arborisation during the perinatal period. Clues to the other targets mediating the neuronal function of FOXP1 could come from FOXP2, since these repressors heterodimerise^20^ and can share promoter occupancy^45^. FOXP2 regulates a network of genes implicated in neurite outgrowth, including genes directly involved in dendrite formation in the cortex, such as the Eph receptor *EphrB2*, and indirectly, like the transcription factor *Lmo4* ^46^.

It is important to note that FOXP1 also functions as a transcriptional repressor outside the nervous system. For example, in the immune system FOXP1 is involved in naïve T-cell quiescence^47^ and B-cell development^48^. Interestingly, stimulation of T-cell antigen receptor signalling regulates the SUMOylation of the kinase PCKθ^49^ and the transcription factor IRF4^50^. This raises the intriguing possibility that TCR signalling in the immune system could be analogous to NMDAR signalling in the nervous system and regulate the SUMOylation of FOXP1 to control its binding to CtBP1 and transcriptional repression.

Together our data suggest SUMO-dependent regulation of FOXP1 is a key component in gene expression programs during activity-dependent remodelling of neuronal architecture and provide new mechanistic insight into how dysregulation of human FOXP proteins can lead to neurodevelopmental disorders such as language impairment and autism spectrum disorders.

## Methods

### Buffers

Modified RIPA: 25 mM Tris-HCl, ph 7.5, 125 mM NaCl, 1% NP40, 0.5% sodium deoxycholate and 1 mM EDTA.

SDS-containing buffer: 1 part SDS sample buffer: 5% SDS, 150 mM Tris-HCl, pH 6.7, 30% glycerol plus 3 parts of RIPA buffer: 25 mM Tris pH 8.2, 50 mM NaCl, 0.5% NP40, 0.5% sodium deoxycholate and 0.1% SDS.

### DNA constructs

A rat-specific shRNA sequence against FOXP1 (5′-TGCGAGTAGAGAACGTTAAA-3′) was cloned into the lentiviral vector pXLG3^51^. shRNA-resistant FOXP1 contained three silent base mismatches in the ORF cloned from E18 rat brain cDNA using the primer: 5-CGA GTG GAA AAC GTT AAA-3′. The resulting ORF was inserted into the same pXLG3 vector, where a separate expression cassette driven by the ubiquitin promoter was inserted to express GFP-IRES-FOXP1. SUMOylation-deficient FOXP1-K670R rescue was generated by site-directed mutagenesis. These constructs were also sub-cloned into the TAP-tagging vector pNTAP-B (Agilent), which tags proteins with both streptavidin-binding peptide (SBP) and calmodulin-binding peptide (CBP) tags to generate TAP-FOXP1, as well as pGEX4T1 (Amersham) and pEGFP-C2 (Clontech).

### Antibodies

Anti-FOXP1 (D35D10, Cell Signaling), anti-EGFR (ab98133, Abcam), anti-Lamin B (Santa Cruz), anti-RhoGDI (EPR3773, Abcam), anti-HDAC2 (3F3, Cell Signaling), anti-HDAC4 (4A3, Cell Signaling), anti-LSD1 (Cell Signaling), anti-Ctbp1 (EMD millipore), anti-SMRT (EMD millipore), anti-GFP (ab13970, abcam), anti-MAP2 (Sigma), anti-GAPDH (Abcam), and anti-SBP (EMD millipore).

### Lentivirus production

Lentiviral particles were produced in HEK293T cells maintained in DMEM (D5796, Sigma) supplemented with 10% (v/v) fetal bovine serum (F7524, Sigma) as previously described^51^. After harvesting, DIV1 rat cultured cortical neurons were incubated with lentiviral particles for 6 hr followed by media change into Neurobasal feeding medium (Thermo Fisher)

### Mass spectrometry

HEK293T cells were transfected with either empty TAP vector or TAP-FOXP1 and lysed 48 hr after transfection in lysis buffer (50 mM Tris-HCl, pH 7.5, 150 mM NaCL, 0.5% NP-40 and Roche protease inhibitor cocktail) before pull-down of the TAP tag using strepavidin beads (Sigma) for 1 hr. The samples were resolved on 4–15% precast gels (BioRad), silver stained and bands specific to the TAP-FOXP1 pulldown only were subjected to liquid chromatography-tandem mass spectrometry analysis on an Orbitrap Velos mass spectrometer (Thermo Fisher) to identify interacting-proteins.

### Bacterial SUMOylation

The bacterial SUMOylation assay was performed as described previously^25^ and the resulting SUMOylated GST-FOXP1 bound to glutathione beads was incubated in equal amounts with or without purified his6-Senp1 catalytic domain to promote deSUMOylation. Immobilised SUMOylated or de-SUMOylated GST-FOXP1 was then incubated with cortical neuronal lysates for 1 hr, followed by extensive washing and subsequent analysis by SDS-PAGE and western blotting.

### Dimerisation assay

HEK293T cells were transfected with either: empty TAP vector, TAP-FOXP1-WT or TAP-FOXP1-K670R along with GFP-FOXP1. Cells were harvested in lysis buffer (0.5% TX-100, 150 mM NaCl, 25 mM Tris pH 7.5 and Roche protease inhibitors) and cleared lysates incubated with strepavidin beads for 1 hr. Samples were washed extensively in lysis buffer and processed for SDS-PAGE and western blotting.

### Cellular fractionation

HEK293T cells transfected with either TAP-WT-FOXP1 or TAP-FOXP1-K670R were fractionated 48 hr after transfection into cytosolic and nuclear fractions using a Cell Fractionation Kit (Cell Signaling) as per the manufacturer’s instructions.

### Transfection and immunocytochemistry

Primary cortical neurons were prepared from Wistar rat E18 embryos as previously described^52^, transfected at DIV1 with Lipofectamine 2000 (ThemroFisher) and fixed 6–7 days later in 4% PFA, before processing for immunocytochemistry and staining.

### SUMOylation analysis

Cultured cortical neurons were lysed in ‘modified’ RIPA without SDS or in SDS-containing buffer containing Roche protease inhibitors. Lysates were incubated on ice for 20 mins, cleared by centrifugation at 13,000 rpm for 10 min, and analysed by SDS-PAGE and western blotting.

### Immunoprecipitation assays

Cells lysed in SDS-sample buffer were diluted 10x with PBS containing 0.5% NP40. After clearing, supernatants were incubated with either non-immune IgG, anti-FOXP1 or anti-SUMO1 antibodies for 2 hours, then with Protein A/G beads, washed and processed for western blotting.

### Senp1-cleavage assay

Washed beads were incubated with either purified free-GST, GST-Senp1-WT or GST-Senp1-C603S proteins in PBS, washed and western blotted.

### Luciferase assay

HEK293 cells were transfected with 50 ng of pGL3-promoter construct (Promega), 50 ng of pRL-TK *renilla* luciferase construct (Promega) and either empty pNTAP-B vector, TAP-tagged FOXP1-WT or TAP-tagged FOXP1-K670R. Cells were lysed 48 hr after transfection and both firefly and renilla luciferase activity quantified using the Dual Luciferase Reporter Assay System (Promega).

#### RT-PCR

Cortical neurons were infected with the indicated lentiviruses for shRNA-mediated knockdown and rescue, followed by lysis and RNA extraction using the RNAeasy kit (Qiagen) as per manufacturers instructions. After subsequent cDNA synthesis, RT-PCR was carried out using standard protocols and PCR cycles were optimised for each reaction so as to be within a linear range. Primers used were as follows: *Foxp1* (5′-TGCGAGTAGAGAACGTTAAA-3′), *CNTNAP2* (CAGCGGAGACACAAACACAT), *gapdh* (5′-AATCCCATCACCATCTTCCA-3′).

### Morphometry of cortical neurons

Images of individual transfected cortical neurons were captured on a Leica SP5 confocal microscope at 20x magnification and at high resolution (1024 × 1024). Acquired images were analysed in ImageJ using the NeuronJ plugin. Two way ANOVA was used followed by Bonferroni multiple comparisons test.
